# Supplementary material for: Correct use and ease-of-use of placebo ELLIPTA dry-powder inhaler in adult patients with chronic obstructive pulmonary disease
Source: PLoS One. 2022 Aug 15;17(8):e0273170. doi: 10.1371/journal.pone.0273170 (PMC9377593; doi:10.1371/journal.pone.0273170)
Supplement: S1 Table — (DOCX) [file pone.0273170.s001.docx]

**Supporting information**

**Table S1.** Ease-of-use questionnaire (ELLIPTA and non-ELLIPTA) for study 200301 [1]

| **Ease-of-use questionnaire** | |
| --- | --- |
| **Question** | **Response options** |
| How do you rate ease-of-use of the inhaler? | Very easy  Easy  Neutral  Difficult  Very difficult |
| How easily are you able to tell how many doses of medication are left in the inhaler? | Very easy  Easy  Neutral  Difficult  Very difficult |
| How do you rate the ease-of-learning how to use the inhaler? | Very easy  Easy  Neutral  Difficult  Very difficult |
| How do you rate the ease-of-handling the inhaler? | Very easy  Easy  Neutral  Difficult  Very difficult |
| How do you rate the ease-of-preparing the inhaler for use? | Very easy  Easy  Neutral  Difficult  Very difficult |
| How do you rate the ease-of-holding the inhaler while using it? | Very easy  Easy  Neutral  Difficult  Very difficult |

**Reference**

1. van der Palen J, Thomas M, Chrystyn H, Sharma RK, van der Valk PD, Goosens M, et al. A randomised open-label cross-over study of inhaler errors, preference and time to achieve correct inhaler use in patients with COPD or asthma: comparison of ELLIPTA with other inhaler devices. NPJ Prim Care Respir Med. 2016;26: 16079.
