# Supplementary material for: Correct use and ease-of-use of placebo ELLIPTA dry-powder inhaler in adult patients with chronic obstructive pulmonary disease
Source: PLoS One. 2022 Aug 15;17(8):e0273170. doi: 10.1371/journal.pone.0273170 (PMC9377593; doi:10.1371/journal.pone.0273170)
Supplement: S2 Table — (DOCX) [file pone.0273170.s002.docx]

**Table S2.** Ease-of-use questionnaire (ELLIPTA) for study 201071 [1].

Instructions: please complete the following questions related to the ELLIPTA inhaler that you used during this study. Choose only one response for each question.

| **Version A** | |
| --- | --- |
| **Question** | **Response options** |
| 1. How easy or difficult is it to use the ELLIPTA inhaler? | Very easy  Easy  Difficult  Very difficult |
| 1. How easy or difficult is it to tell how many doses are left in the ELLIPTA inhaler? | Very easy  Easy  Difficult  Very difficult |
| 1. If your **current daily inhaled asthma/COPD medication^a^** was available in the ELLIPTA inhaler, how likely or unlikely would you be to request the medication in the ELLIPTA inhaler from your doctor? | Very likely  Likely  Unlikely  Very unlikely |
| **Version B** | |
| **Question** | **Response options** |
| 1. How easy or difficult is it to use the ELLIPTA inhaler? | Very difficult  Difficult  Easy  Very easy |
| 1. How easy or difficult is it to tell how many doses are left in the ELLIPTA inhaler? | Very difficult  Difficult  Easy  Very easy |
| 1. If your **current daily inhaled asthma/COPD medication^a^** was available in the ELLIPTA inhaler, how likely or unlikely would you be to request the medication in the ELLIPTA inhaler from your doctor? | Very unlikely  Unlikely  Likely  Very likely |

^a^As relevant; asthma medication for subjects in the asthma study and COPD medication for subjects in the COPD study.

COPD, chronic obstructive pulmonary disease.

**Reference**

1. Feldman GJ, Galkin DV, Patel P, Collison KA, Sharma R. Correct use and ease of use of a placebo dry powder inhaler in subjects with asthma and chronic obstructive pulmonary disease. Chron Respir Dis. 2019;16: 1479973118815692.
