# Supplementary material for: Correct use and ease-of-use of placebo ELLIPTA dry-powder inhaler in adult patients with chronic obstructive pulmonary disease
Source: PLoS One. 2022 Aug 15;17(8):e0273170. doi: 10.1371/journal.pone.0273170 (PMC9377593; doi:10.1371/journal.pone.0273170)
Supplement: Attachment — Submitted filename: 212146_PLOS ONE_Response to Reviewers_29Mar2022STC.docx [file pone.0273170.s003.docx]

Renu Jain

GlaxoSmithKline plc.

Research Triangle Park

Durham

North Carolina

United States

Manlio Milanese

Academic Editor, *PLOS ONE*

29 March 2022

Dear Dr Milanese,

On behalf of my co-authors, I would like to thank you for considering our manuscript for publication and providing us with the opportunity to address comments and make minor revisions suggested by the Editorial Team and Reviewers. Please find clean and tracked changes versions of our revised manuscript enclosed, and our point-by-point replies to the comments below in blue italicised font, where additions have been made bold and deletions have been crossed out. Please note that page and line numbers refer to the version of the manuscript with tracked changes. I hope you will now consider our article suitable for publication in *PLOS ONE*. Please do let me know if anything further is required. We look forward to receiving your decision.

Yours sincerely,

Renu Jain

GlaxoSmithKline plc.

**Journal Requirements:**

When submitting your revision, we need you to address these additional requirements.

1. Please ensure that your manuscript meets PLOS ONE's style requirements, including those for file naming. The PLOS ONE style templates can be found at

https://journals.plos.org/plosone/s/file?id=wjVg/PLOSOne_formatting_sample_main_body.pdf and

https://journals.plos.org/plosone/s/file?id=ba62/PLOSOne_formatting_sample_title_authors_affiliations.pdf

We have reviewed the templates and can confirm our manuscript adheres to PLOS ONE’s style requirements

2. Thank you for stating the following financial disclosure:

This work was funded by GlaxoSmithKline plc. (study 212146).

Please state what role the funders took in the study. If the funders had no role, please state: "The funders had no role in study design, data collection and analysis, decision to publish, or preparation of the manuscript."

If this statement is not correct you must amend it as needed.

Please include this amended Role of Funder statement in your cover letter; we will change the online submission form on your behalf.

We have removed the funding statement from the manuscript (page 25, lines 370–371). Please include the following statement in the online submission form:

*“This work was funded by GlaxoSmithKline plc. (Study 212146). The funders had a role in study design, data collection and analysis, decision to publish, and preparation of the manuscript.”*

3. Thank you for stating the following in the Acknowledgments/ Funding Section of your manuscript:

This work was funded by GlaxoSmithKline plc. (study 212146).

Please note that funding information should not appear in the Acknowledgments section or other areas of your manuscript. We will only publish funding information present in the Funding Statement section of the online submission form.

Please remove any funding-related text from the manuscript and let us know how you would like to update your Funding Statement. Currently, your Funding Statement reads as follows:

This work was funded by GlaxoSmithKline plc. (study 212146).

Please include your amended statements within your cover letter; we will change the online submission form on your behalf.

Thank you for highlighting this. We have removed the funding statement from the manuscript (page 25, lines 370–371) and have included the amended wording above in Comment 2.

4. Thank you for stating the following in the Competing Interests section:

I have read the journal’s policy and the author of this manuscript have the following competing interests: RJ, KC, RS, LS, and JR report employment with, and stock/share ownership in, GlaxoSmithKline plc. during study conduct. KC is currently employed by AstraZeneca and LS is no longer employed by GlaxoSmithKline plc. TMS received research support from West-Ward Pharmaceuticals, Theravance Biopharma US, Inc., GlaxoSmithKline plc., Pearl Therapeutics, Chiesi, AstraZeneca, Novartis, Boehringer Ingelheim, Forest, Compleware, Evidera, Oncocyte, Teva, Vapotherm, Sunovion, Proterix BioPharma, Seer, and Sanofi. TMS has also received speaker fees from GlaxoSmithKline plc., Mylan Inc./Theravance Biopharma US, Inc., and Sunovion, and consulting fees from Vapotherm. DIB received grant/research/clinical trial support from GlaxoSmithKline plc., Teva, AstraZeneca, Pearl Therapeutics, Novartis, Genentech, Inc., Merck, Boehringer Ingelheim, Amgen, Aimmune, Shire, and Biocryst and consulted/participated in advisory boards for GlaxoSmithKline plc., ALK America, Gerson-Lehman, and Guidepoint Global.

Please confirm that this does not alter your adherence to all PLOS ONE policies on sharing data and materials, by including the following statement: "This does not alter our adherence to PLOS ONE policies on sharing data and materials.” (as detailed online in our guide for authors http://journals.plos.org/plosone/s/competing-interests). If there are restrictions on sharing of data and/or materials, please state these. Please note that we cannot proceed with consideration of your article until this information has been declared.

Please include your updated Competing Interests statement in your cover letter; we will change the online submission form on your behalf.

We have included the following in the revised manuscript:

Author disclosure statements, page 25, lines 355–368: *“RJ, KC, RS, LS, and JR report employment with, and stock/share ownership in, GlaxoSmithKline plc. during study conduct. KC is currently employed by AstraZeneca and LS is no longer employed by GlaxoSmithKline plc. TMS received research support from West-Ward Pharmaceuticals, Theravance Biopharma US, Inc., GlaxoSmithKline plc., Pearl Therapeutics, Chiesi, AstraZeneca, Novartis, Boehringer Ingelheim, Forest, Compleware, Evidera, Oncocyte, Teva, Vapotherm, Sunovion, Proterix BioPharma, Seer, and Sanofi. TMS has also received speaker fees from GlaxoSmithKline plc., Mylan Inc./Theravance Biopharma US, Inc., and Sunovion, and consulting fees from Vapotherm. DIB received grant/research/clinical trial support from GlaxoSmithKline plc., Teva, AstraZeneca, Pearl Therapeutics, Novartis, Genentech, Inc., Merck, Boehringer Ingelheim, Amgen, Aimmune, Shire, and Biocryst and consulted/participated in advisory boards for GlaxoSmithKline plc., ALK America, Gerson-Lehman, and Guidepoint Global.* ***This does not alter our adherence to PLOS ONE policies on sharing data and materials.^”^***

5. In your Data Availability statement, you have not specified where the minimal data set underlying the results described in your manuscript can be found. PLOS defines a study's minimal data set as the underlying data used to reach the conclusions drawn in the manuscript and any additional data required to replicate the reported study findings in their entirety. All PLOS journals require that the minimal data set be made fully available. For more information about our data policy, please see http://journals.plos.org/plosone/s/data-availability.

Upon re-submitting your revised manuscript, please upload your study’s minimal underlying data set as either Supporting Information files or to a stable, public repository and include the relevant URLs, DOIs, or accession numbers within your revised cover letter. For a list of acceptable repositories, please see http://journals.plos.org/plosone/s/data-availability#loc-recommended-repositories. Any potentially identifying patient information must be fully anonymized.

Important: If there are ethical or legal restrictions to sharing your data publicly, please explain these restrictions in detail. Please see our guidelines for more information on what we consider unacceptable restrictions to publicly sharing data: http://journals.plos.org/plosone/s/data-availability#loc-unacceptable-data-access-restrictions. Note that it is not acceptable for the authors to be the sole named individuals responsible for ensuring data access.

We will update your Data Availability statement to reflect the information you provide in your cover letter.

We have updated the Data Availability statement as follows:

Data Availability statement, page 24, lines 348–351: *“~~Anonymized individual participant data and study documents can be requested for further research from www.clinicalstudydatarequest.com.~~*

***The authors confirm that the data supporting the findings of this study are available within the article and its supplementary materials.^”^***

6. PLOS requires an ORCID iD for the corresponding author in Editorial Manager on papers submitted after December 6th, 2016. Please ensure that you have an ORCID iD and that it is validated in Editorial Manager. To do this, go to ‘Update my Information’ (in the upper left-hand corner of the main menu), and click on the Fetch/Validate link next to the ORCID field. This will take you to the ORCID site and allow you to create a new iD or authenticate a pre-existing iD in Editorial Manager. Please see the following video for instructions on linking an ORCID iD to your Editorial Manager account: https://www.youtube.com/watch?v=_xcclfuvtxQ

Thank you for this request. We have added the following ORCID details to the title page of the manuscript:

Title page, page 1, lines 19–21: *“****ORCID details***

***Thomas M. Siler 0000-0002-5103-0360***

***Renu Jain 0000-0001-5400-2536***”

Please may we kindly advise that the YouTube link in the above comment is linked to a private video, so the content cannot be accessed.

7. Please review your reference list to ensure that it is complete and correct. If you have cited papers that have been retracted, please include the rationale for doing so in the manuscript text, or remove these references and replace them with relevant current references. Any changes to the reference list should be mentioned in the rebuttal letter that accompanies your revised manuscript. If you need to cite a retracted article, indicate the article’s retracted status in the References list and also include a citation and full reference for the retraction notice.

We have reviewed the reference list and can confirm it is complete, up to date and correct, with no retracted references.

**Additional Editor Comments:**

This retrospective study on pooled data coming from 4 studies assessing use of Ellipta DPI in COPD patients is of clinical interest and well-written. Please, answer point by point to the two reviewers to increase the quality of the manuscript.

**Reviewers' Comments to the Author:**

**Reviewer #1**

This is a retrospective study on pooled data coming from 4 studies assessing use of Ellipta DPI in COPD patients. The paper is of interest for the readers of the Journal and results sound. I have the following question that the Authors need to address.

We thank the Reviewer for their kind words about our manuscript.

Scrutiny of the figure 3 seems to indicate that there is a significant (and not a trend as reported in the text) difference in the % of patients using Ellipta correctly between patients aged 40-64 and those older than 75 years (see CIs between groups). Please check it with appropriate statistical methods.

Thank you for this query. As there were no statistical analyses performed on the data, it is not possible to state if the results were significant. We have added the following to the results and discussion sections as follows:

Results, page 14, lines 184–192: “*Correct use (Fig 3) and user ratings of “easy” or “very easy” (Fig 3) of the placebo ELLIPTA DPI remained high with increasing age, including patients aged ≥65 years. However, among the ≥75 years age group, there was a slight trend towards a decrease in the proportion of patients demonstrating correct use of the placebo ELLIPTA DPI at study end and in the proportion finding its use “very easy” (Fig 3, 4).* ***The 95% CI for the 40-64 age group’s correct use do not overlap with the
65–74 and 75+ year groups, suggesting significant differences – however no statistical modelling was performed to investigate the differences in age groups.***”

Discussion, page 22, lines 302–306: *“However, the data were not truly homogenous, and the level of heterogeneity was not assessed****.*** ***For the investigation of age groups, the 95% confidence intervals between the 40–64 years age group do not overlap with the other two age ranges, suggesting a significant difference in proportions, however no statistical tests comparing the differences between age groups were performed****.”*

**Reviewer #2**

I would like to congratulate the authors for this interesting and well-written work. I have just a few comments that should be addressed in the manuscript.

We thank the Reviewer for their kind words about our manuscript.

The secondary endpoint was the percentage of patients rating the placebo

ELLIPTA DPI as “easy” or “very easy” to use at study end, as assessed by

questionnaires in patients deemed to be using the placebo ELLIPTA DPI correctly at

study end. What does it mean? Did the Authors consider only question 1 of the questionnaires?

Thank you for this query. We can confirm that only question 1 of the questionnaires was considered as part of this analysis, and further analyses might consider the remaining questions that were posed (ability to tell how many doses remained, likelihood of asking for ELLIPTA if their medications were available in the inhaler). We believe that the meaning of this result is that patients find it easy to use the inhaler, regardless of prior experience with it. This result is supported by the literature, and the meaning of this result and its context in the wider literature is included in the discussion section as follows:

Discussion, page 19, lines 241–245: *“Correct placebo ELLIPTA DPI use, and placebo ELLIPTA DPI use that was rated “easy” or “very easy”, both remained high in patients aged ≥65 years, suggesting that use of the ELLIPTA DPI would provide appropriate drug delivery to the lungs and would subsequently increase the likelihood of successful clinical outcomes in this patient population.”*

Discussion, page 20, lines 257–267: *“In a study of responses of patients with COPD for the Global Usability Score questionnaire (developed for assessing and comparing the usability of different inhalation devices simultaneously), the ELLIPTA inhaler was identified as having the highest usability score among tested DPIs (Breezhaler, DISKUS, Genuair, Nexthaler, Spiromax, and Turbohaler). This score was independent of the patient’s level of DPI experience. The ELLIPTA inhaler was also rated as the quickest to learn and requiring the shortest time for patients to achieve autonomy [28]. Similarly, in a study comparing first experiences with different inhalers in COPD (pMDI, Aerolizer, HandiHaler, Turbohaler, DISKUS, Breezhaler, ELLIPTA, Easyhaler, DISKHALER and Respimat), most participants were able to use the ELLIPTA inhaler correctly with ≤1 counseling attempts [29].”*

However, there were several limitations in the study designs regarding the use of the ELLIPTA inhaler which are noted in the discussion section:

Discussion, page 21, lines 283–291: *“At study level, a limitation of study 201071 [16] was that screening procedures involved an assessment of correct inhaler use, although few patients were excluded for this reason. Study 201071 [18] allowed recruitment of patients who had not used the ELLIPTA inhaler within 6 months, study 206901 [20] within 12 months and study 206215 [19] within 24 months prior to screening, while study 200301 [17] included only patients who were completely naïve to the inhaler. Therefore, the possibility that the correct ease-of-use data in studies 201071 [18], 206901 [20], and 206215 [19] were influenced by the patients’ previous experience with the ELLIPTA inhaler cannot be discounted.”*

The secondary outcome has been assessed by mean of unvalidated tools. This is something that should be discussed. The use of unvalidated questionnaires to evaluate the patient reported outcomes is a limit.

We thank the Reviewer for highlighting this to us. We have added the following to the discussion section:

Discussion, page 22, lines 302–308: *“However, the data were not truly homogenous, and the level of heterogeneity was not assessed****. For the investigation of age groups, the 95% confidence intervals between the 40–64 years age group do not overlap with the other two age ranges, suggesting a significant difference in proportions, however no statistical tests comparing the differences between age groups were performed****.* ***It should also be noted that the use of unvalidated questionnaires to evaluate the secondary patient-reported outcomes is another limitation of the study.”***
